# Swallowing Gel for Patients with Dysphagia: A Novel Application of Chitosan

**DOI:** 10.3390/gels7030108

**Published:** 2021-08-05

**Authors:** Tanikan Sangnim, Pornsak Sriamornsak, Inderbir Singh, Kampanart Huanbutta

**Affiliations:** 1 Faculty of Pharmaceutical Sciences, Burapha University, 169 Longhaad Bangsaen Road, Saensuk, Mueang, Chon Buri 20131, Thailand; tanikan@go.buu.ac.th; 2 Department of Pharmaceutical Technology, Faculty of Pharmacy, Silpakorn University, Nakhon Pathom 73000, Thailand; sriamornsak_p@su.ac.th; 3 Academy of Science, The Royal Society of Thailand, Bangkok 10300, Thailand; 4 Chitkara College of Pharmacy, Chitkara University, Patiala 140401, Punjab, India; inderbir.singh@chitkara.edu.in

**Keywords:** chitosan, geriatric patients, pill swallowing gel, dysphagia

## Abstract

Dysphagia refers to difficulty swallowing certain foods, liquids, or pills. It is common among the elderly with chronic diseases who need to take drugs for long periods. Therefore, dysphagia might reduce compliance with oral drug administration in the aging population. Many pharmaceutical companies search for new products to serve as swallowing aids. Existing products are expensive and do not suit all geriatric patients. Therefore, this study aimed to develop and investigate pill swallowing aid gels prepared from carboxymethyl cellulose and chitosan. We formulated gels by dissolving different concentrations of carboxymethyl cellulose and low or high molecular weight chitosan in solvents to find appropriate gel rheology properties. We then added several portions of glycerin as the glidant of the formulation. We found that the optimized gel formulation was 6.25% (*w*/*w*) chitosan with a molecular weight of 80–120 kDa dissolved in 1.2% acetic acid and 4% (*w*/*w*) glycerin. The developed pill swallowing gel’s rheology was pseudoplastic with a viscosity of 73.74 ± 3.20 Pa⸱s. The developed chitosan gel had enhanced flow ability; it allowed the pill to cross a 300 mm tube within 6 s, while the reference product took 3 s. Even though the reference product could carry the pill in the tube faster, the chitosan gel better covered the pill, making it more convenient to use. Finally, using a theophylline tablet as a model tablet dosage form, we assessed the gel’s effect on drug disintegration and dissolution. The chitosan gel delayed the tablet disintegration time by about 3–7 min and slightly affected the theophylline dissolution rate. Lastly, all gels were physically stable after a month of storage in the stress condition. These results show the feasibility of manufacturing a chitosan gel usable as a pill swallowing gel for patients with dysphagia.

## 1. Introduction

In industrialized countries, increasing life expectancy and declining birth rates are shifting the age distribution of populations toward older age [1]. With increasing age comes chronic diseases; therefore, geriatric patients often require several daily drugs (polypharmacy) [2]. This leads to medication errors and treatment failure. Moreover, many physiological changes affect the pharmacokinetics of drugs in the aging population [3]. One of the critical issues of geriatric patients is the difficulty swallowing solid dosage forms called dysphagia.

Consequently, novel dosage forms designed for older adults and medical devices/tools have emerged in the last decades, such as oral disintegrating tablets [4,5], composite freeze dried wafers [6,7], spray solution for tablet/capsule film coating [8], and spray solution for oral cavity films [9]. Nonetheless, novel dosage forms can be time-consuming and expensive, and other swallowing aid innovations are inconvenient. Finally, pill swallowing gels are an option in oral drug administration of solid dosage forms.

Pill swallowing gel (PSG) products have advantages over novel dosage forms. They can be used with regular tablets/capsules at an affordable cost and mask an eventual unpleasant taste. Moreover, PSGs preserve drug release kinetics since the medication is not required to be crushed before administration. PSGs can also protect the gastrointestinal mucosa, particularly the esophagus, and reduce esophagitis [10]. PSGs work by simply covering the tablets/capsules. They reduce friction and tension during the swallowing process through the pharynx and esophagus [11]. They can also increase the tablet weight and thereby aid passage down the esophagus into the stomach. Several PSG products use hydrophilic polymers such as hydroxypropylmethylcellulose [12] as a gel-forming agent. Most of the used polymers in the PSGs are synthetic which might cause more toxic than natural ones.

Several biopolymers, like alginates, carrageenan, pectin, gum, and chitosan [13], have served as gelling agents in pharmaceutical formulations. They indeed hold various advantages over other synthetic polymers: They are less expensive, non-toxic, and readily available [14]. Chitosan has multiple uses in drug delivery [15,16,17] since it offers low toxicity (both in its native and salt forms) [18], has good swelling properties [19], and can be obtained from food industry waste [20]. The chitosan structure contains an amine group on each deacetylated unit that is protonated in acidic conditions [21]. Thus, concentrated aqueous solutions of chitosan in acidic conditions result in gel formation. The gel-forming properties and biocompatibility of chitosan show the high feasibility of applying chitosan as a novel gel-forming agent in PSG. However, the preparation parameters and other excipients are necessary to optimize for practical PSG formulation.

In the present study, we aimed to develop a PSG from chitosan. To find a PSG with suitable viscosity and rheology properties, we varied chitosan molecular weights (low and high), chitosan concentration, chitosan/acid ratio, and the amount of additive. We compared the chitosan PSG with a PSG prepared from the synthetic polymer carboxymethyl cellulose. The effect of the chitosan PSG on drug dissolution was assessed to confirm that the product would not affect drug absorption and drug concentration in plasma.

## 2. Results and Discussion

### 2.1. Physical Appearance and Rheology

**Figure 1:**
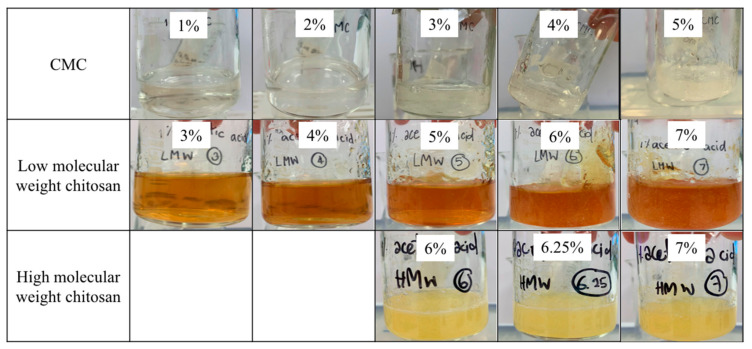
Physical appearance of the gels prepared from different concentrations of carboxymethyl cellulose (CMC), low molecular weight chitosan, and high molecular weight chitosan.

**Figure 2:**
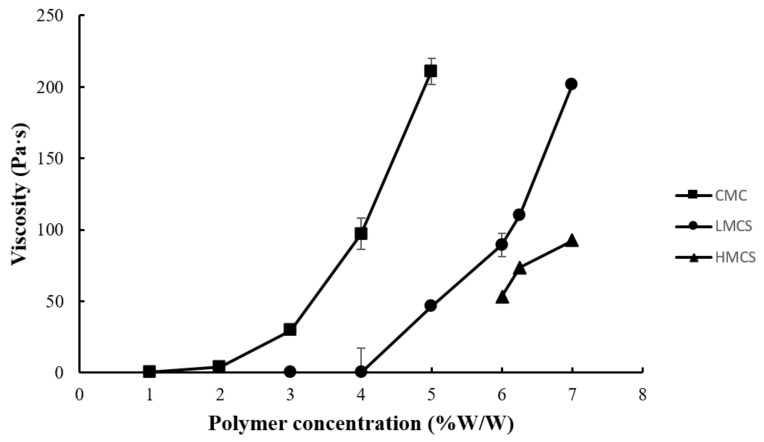
Viscosity at shear rate of 1 s^−1^ of the gels prepared from different concentrations of carboxymethyl cellulose (CMC), low molecular weight chitosan (LMCS), and high molecular weight chitosan (HMCS).

Figure 1 presents the physical appearances of the gels prepared from carboxymethyl cellulose (CMC), low molecular weight chitosan (LMCS), and high molecular weight chitosan (HMCS). The polymer concentrations were screened to find gels with suitable properties. We selected polymer concentration ranges based on visual observation of viscosity and clarity. Gels with low polymer concentrations lacked viscosity, and those with high polymer concentrations did not flow even when tilting the beaker at more than 90°. For the viscosity measurements using a rheometer, we selected the concentration ranges 1–5%, 3–7%, and 6–7% for CMC, LMCS, and HMCS, respectively. As shown in Figure 2, the measured viscosity (shear rate 1 s^−1^) of all the polymer gels in the selected concentration ranges was between 0.011 and 210.767 Pa s. At a similar polymer concentration, the HMCS gel had a higher viscosity than LMCS and CMC ones.

**Figure 3:**
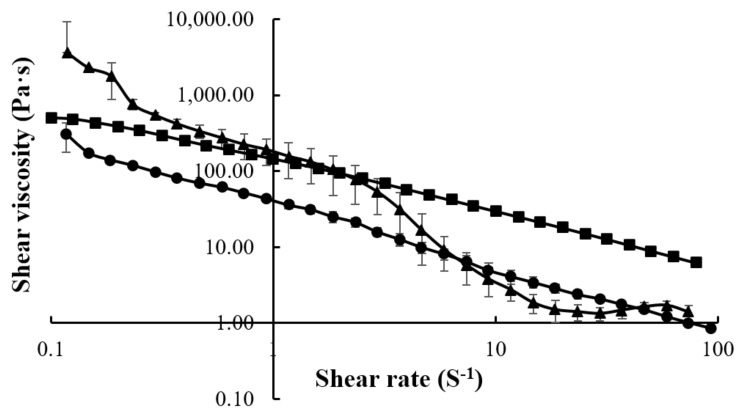
Flow curves of the gels prepared from (■) 4% carboxymethyl cellulose (CMC), (●) 5% low molecular weight chitosan (LMCS), and (▲) 6.25% high molecular weight chitosan (HMCS).

The CMC, LMCS, and HMCS concentrations of 4%, 5%, and 6.25% were selected from the gel viscosity screening results, respectively, to evaluate rheology behavior. Indeed, these gels had viscosities of about 50–100 Pa·s which is suitable for patients with dysphagia, according to a previous study [22]. Figure 3 shows the flow curves of the gels prepared from 4% CMC, 5% LMCS, and 6.25% HMCS. All the displayed shear thinning is the non-Newtonian behavior of fluids, the viscosity of which decreases under shear strain [23]. At the resting state, the HMCS gel had a higher viscosity than the LMCS and CMC gels. Nevertheless, when the shear rate increased, the shear viscosity of HMCS dropped lower than the others.

**Figure 4:**
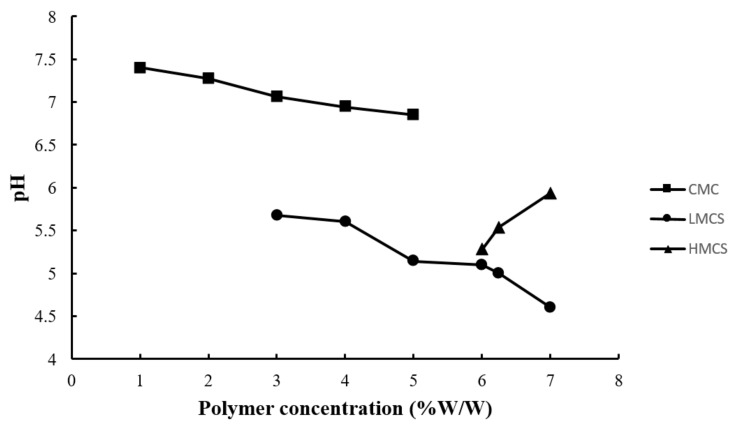
pH of the gels prepared from different concentrations of carboxymethyl cellulose (CMC), low molecular weight chitosan (LMCS), and high molecular weight chitosan (HMCS).

Figure 4 exhibits the pH of the prepared gels. All the pH values were between 4.60 and 7.40, within the range of edible pH, and should not cause irritation [24]. The gels prepared from chitosan (low and high molecular weight) had a lower pH than those from CMC. This came from the acetic acid used to solubilize chitosan [19].

**Figure 5:**
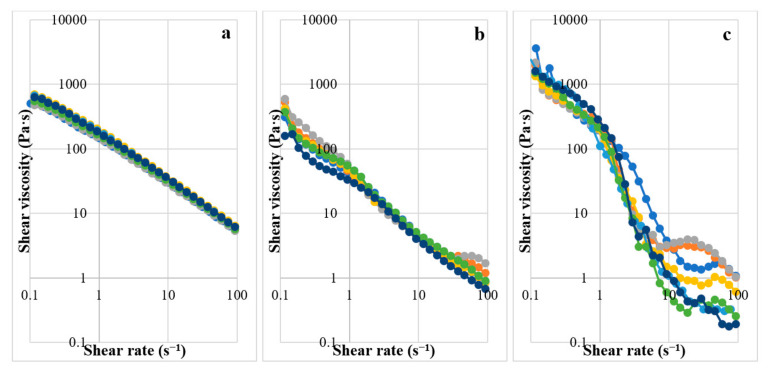
Flow curves of the gels prepared from (**a**) 4% carboxymethyl cellulose (CMC), (**b**) 5% low molecular weight chitosan (LMCS), and (**c**) 6.25% high molecular weight chitosan (HMCS) with different glycerin concentrations (1–7%).

**Figure 6:**
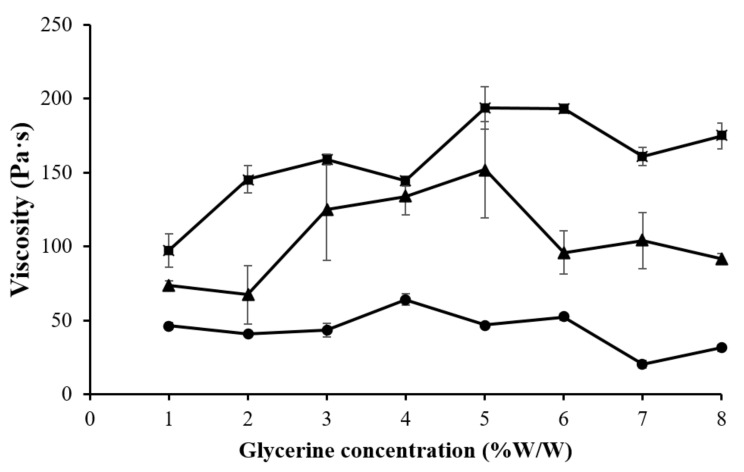
Viscosity of the gels prepared from (■) 4% carboxymethyl cellulose (CMC), (●) 5% low molecular weight chitosan (LMCS), and (▲) 6.25% high molecular weight chitosan (HMCS) with different glycerin concentrations.

The gels prepared from 4% CMC, 5% LMCS, and 6.25% HMCS were selected for further formulation development. Glycerin at different concentrations (1–7% *w*/*w*) was added to the PSG formulation to enhance gel flow. Figure 5 displays the PSG flow curves. The HMCS gel had a higher shear viscosity in the resting state than the CMC and LMCS gels. However, at high shear rates (50–100 s^−1^), the HMCS gel had a lower viscosity. From the flow curves, we could not observe the effect of the different glycerin concentrations. Consequently, we picked and plotted the viscosity of the 4% CMC, 5% LMCS, and 6.25% HMCS gels at the shear rate of 10 s^−1^ at different glycerin concentrations (Figure 6). According to the graph, adding 5% of glycerin to the HMCS and CMC gels significantly increased the viscosity compared with those without glycerin, while glycerin did not alter the viscosity of the LMCS gel. This might be because adding a suitable amount of glycerin into the hydrophilic polymer solutions can generate interactions of hydrogen bonds between polymer and glycerin, increasing the viscosity of the solutions. However, the interaction of the hydrogen bonds can be inhibited by excessive glycerin addition. This is because the saturated glycerin portion has the effect of diluting the solution [25].

### 2.2. Flowability of the Gels

#### 2.2.1. Gel Flow on a Tilted Aluminum Plate

**Figure 7:**
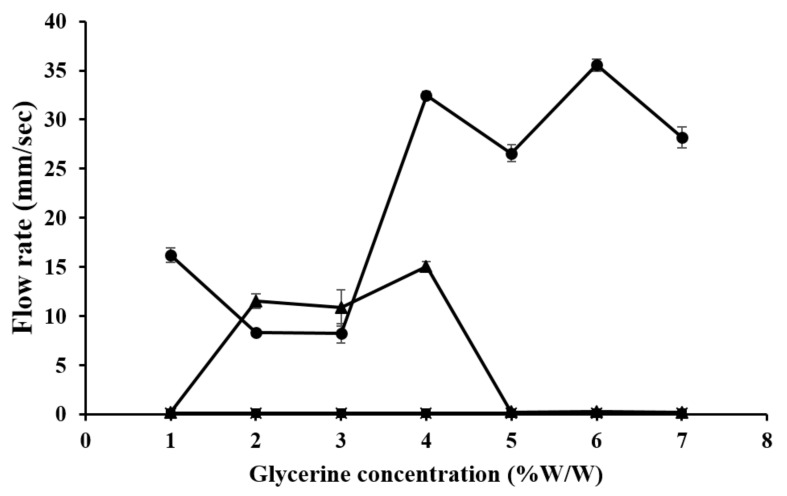
Flow rate on a tilted aluminum plate of gels prepared from (■) 4% carboxymethyl cellulose (CMC), (●) 5% low molecular weight chitosan (LMCS), and (▲) 6.25% high molecular weight chitosan (HMCS) with different glycerin concentrations.

We performed the tilted-aluminum-plate test on the gels prepared from 4% CMC, 5% LMCS, and 6.25% HMCS with different glycerin concentrations (1–7% *w*/*w*). As illustrated in Figure 7, glycerin did not accelerate gel flow in the CMC gel, but some concentrations could significantly raise the flow rate of the LMCS and HMCS gels. In the LMCS gel, 4–7% glycerin increased the flow rate (26.58–35.56 mm/s). This might be because glycerin can modify the hydrogen bonding between water and the polymer, thereby affecting the swelling and viscoelastic properties of the polymers. In an aqueous solvent, the polymer favors interactions with the solvent over intramolecular interactions. Thus, the polymer chains are well expanded [26]. Flow rate of the HMCS gels dropped at the glycerin concentration of 5–7% (*w*/*w*). This is due to the fact that excessive glycerin concentration raises mixed gel viscosity, resulting in delaying of gel flow. This result is in agreement with the previous work by Ayala et al. [27].

#### 2.2.2. Tablet Carrying through a Tube

**Figure 8:**
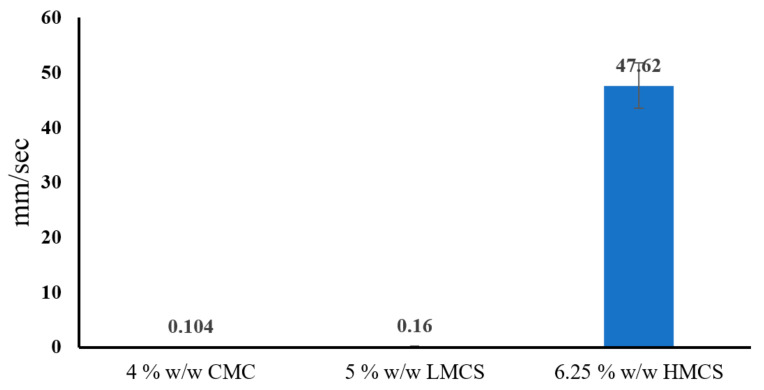
Flow rate of the selected pill swallowing gel formulations from the tablet-through-a-tube test (carboxymethyl cellulose; CMC, low molecular weight chitosan; LMCS, and high molecular weight chitosan; HMCS).

According to the gel flow on a tilted aluminum plate result (Figure 7), glycerin concentrations at 1, 6, and 4 offering high flow rate in CMC, LMCS, and HMCS gels were selected to formulate the PSGs for the tablet-through-a-tube test, as indicated in Table 1. Figure 8 shows the flow rates of all the selected formulations. The HMCS gel had a significantly faster flow rate than the LMCS and CMC gels. This might be because the HMWC gel had a higher weight than the other gels at a similar volume. Therefore, the tablet covered with PSG-HCS could travel faster vertically. This result is also in agreement with the rheology test. As the flow curves of the HMCS gel in Figure 5 show, the viscosity decreases at higher shear rates. The movement of the gel or liquid through the tube increases the shear rate of about 10–10^3^ s^−1^ [28]. Consequently, the HMCS gel has a lower viscosity and faster flow than the other polymer when passing through the tube.

### 2.3. PSG Effect on Tablet Disintegration

Table 1 reports the disintegration time of the theophylline plain tablet and the tablet covered with different PSG formulations. The plain tablet disintegration time was 3.51 ± 0.30 min, faster than with the CMC, LCS, and HCS gels. This is because all the gels are hydrophilic and can delay the penetration of the medium (distilled water) into the tablet. The result is consistent with a previous study by Takashi Tomita and colleagues. It reported that a PSG prepared from food thickener extended tablet disintegration time from 27 s (plain levofloxacin tablet) to about 195–608 s [29]. The disintegration time of the tablet enveloped with the HMCS gel was faster than that of tablets covered with the LMCS and CMC gels. This can be explained by the flow curves shown in Figure 5. According to the flow curve, the HMCS gel rheology behavior is shear thinning [30]. This makes the HMCS gel viscous at the beginning (low shear rate). When the disintegration basket rack moved, it increased the shear rate and thus dramatically reduced the viscosity of the gel, liberating the tablet.

### 2.4. PSG Effect on Drug Dissolution

**Figure 9:**
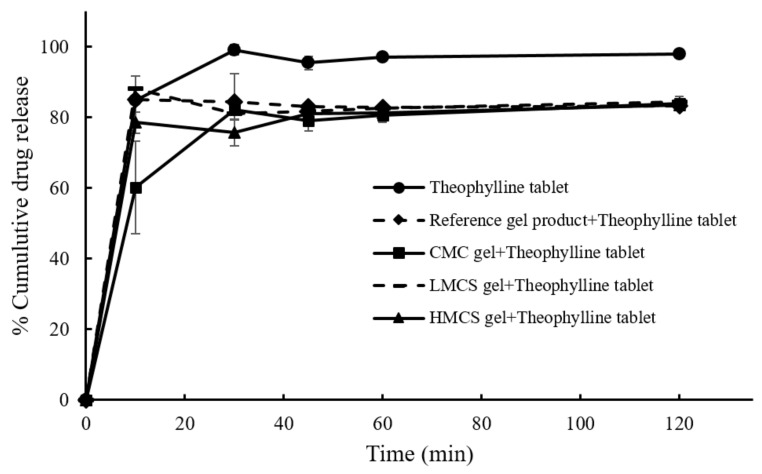
Dissolution profiles of the theophylline tablets and the tablet covered with different pill swallowing gel formulations.

Figure 9 displays the dissolution profiles of the theophylline plain tablet and the tablet covered with the different PSG formulations and the reference PSG commercial products. For all the conditions, the drug dissolution reached a maximum of around 30 min. According to the theophylline tablet monograph in USP40, the plain tablet passed the dissolution test, but the PSG-covered ones did not meet the 85% (Q + 5) cumulative drug release criteria at 45 min [31]. The tablets covered with PSGs (all the formulations) and the reference product all had a slower drug release than the plain tablet. Considering Table 2, the dissimilarity factors (*f*_2_) of the reference product and LMCS gel-covered theophylline tablet were not within the accepted limit (0–15). None of the test samples were within the similarity factor (*f*_1_) accepted range (50–100). The tablet’s dissolution difference factors (*f*_1_ − *f*_2_) covered with the reference and LMCS gel were very close to that of the plain tablet. These results indicate that the PSGs from this study and the reference product delayed drug release. This agrees with the previous study by Takashi Tomita [29], reporting that the disintegration time of the food-thickener-covered tablet was twice that of the plain tablet. Moreover, the food thickener delayed the dissolution of the model drug (levofloxacin) by about 100 min. However, the oral administration of plain tablets or food-thickener-covered tablets to human subjects (four healthy Japanese adult men) revealed no significant difference in plasma levofloxacin concentration at 15 and 180 min. This might be because gastrointestinal fluid and peristalsis washed out the gel.

### 2.5. Stability of PSG

**Figure 10:**
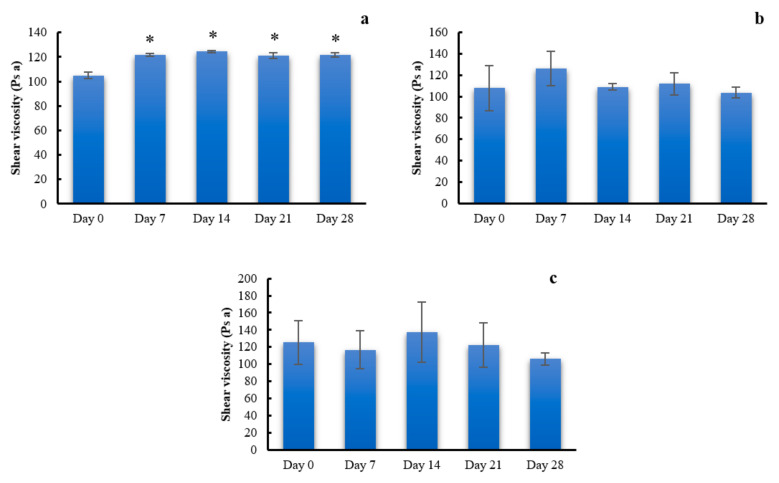
Viscosity of the gels prepared from (**a**) 4% carboxymethyl cellulose (CMC), (**b**) 5% low molecular weight chitosan (LMCS), and (**c**) 6.25% high molecular weight chitosan (HMCS) from the stress condition stability tests. (* indicates a *p* value less than 0.05).

Viscosity of the PSG with glycerin prepared from 4% CMC, 5% LMCS, and 6.25% HMCS from the stress condition stability tests is depicted in Figure 10. It was found that viscosities of the PSG prepared from CMC were slightly but significantly higher than at the start date (Figure 10a). However, viscosities of PSG from LMCS and HMCS were physically stable under the stress condition for 28 days (Figure 10b,c). The *p* values from one-way ANOVA were 0.1032 and 0.7016 for LMCS and HMCS-PSG, respectively. This is might be due to water loss from CMC-PSG resulting in more viscous solution [32], while the LMCS and HMCS-PSG contain higher glycerin concentration, which can protect water evaluation during the stability test through the water sorption mechanism [33].

## 3. Conclusions

Low and high molecular weight chitosan could be used as a gelling agent in a PSG to enhance the swallowing ability of patients with dysphagia. The rheology of the chitosan and CMC gels revealed a shear thinning behavior, which is suitable for PSG products due to the shear viscosity reduction obtained by applying a simulated swallowing force. The HMCS at a concentration of 6.25% with 4% glycerin offered efficient tablet-carrying flow through the tube. The in vitro dissolution profiles of the PSG-covered tablets were slightly slower than that of the plain theophylline tablet.

## 4. Materials and Methods

### 4.1. Materials

Chitosan with low (30–80 kDa, deacetylation percent of 93% (lot no. L112-200330/01)) and high (80–120 kDa, deacetylation percent of 93% (lot no. L112-200330/01)) molecular weights were purchased from BIO21 (Chonburi, Thailand). CMC (lot no. 73H0365) was obtained from Sigma Aldrich, St. Louis, MO, USA. The commercial theophylline tablets (125 mg) were received from Westmont, Bangkok, Thailand (lot no. AM21N006). All other chemicals were of standard pharmaceutical grade.

### 4.2. Chitosan and CMC PSG Preparation

The chitosan PSGs were prepared by dissolving low molecular weight chitosan (LMCS) and high molecular weight chitosan (HMCS) at a concentration of 1–7% (*w*/*w*) in a 1.0% acetic acid solution and stirring for 12 h. To prepare the CMC PSG, we dissolved various concentrations of CMC (1–7% (*w*/*w*)) in distilled water. After obtaining suitable LMCS, HMCS, and CMC concentrations, we added a different portion of glycerin as a lubricant.

### 4.3. PSG Evaluation

#### 4.3.1. Physical Appearance, Rheology, and Viscosity

The prepared gels were photographed, and their physical appearance, including clarity, precipitate, and viscosity, was observed. We then measured the rheological behaviors of the CMC, LMCS, and HMCS using the inflow curve mode (viscosity vs. shear rate) of a rheometer (KINEXUS Lab+, MS603S/01, Malvern Panalytical Ltd., Malvern, UK). We performed the measurements at a controlled temperature (25 °C), set the shear rate range to 0.1–10 Pa·s, and selected the viscosity at a shear rate of 1 s^−1^ as a representative of the formulation. The experiments were carried out in triplicate. All prepared PSGs were also kept at 50 °C and 75% relative humidity (RH) for stability evaluation (stress testing).

#### 4.3.2. pH of the Gels

The pH of the PSGs was measured to make sure that the prepared product would not cause irritation. The sample was prepared by dissolving 5 g of the gels in 3 mL of distilled water then measured the pH of the solution with a pH meter (SevenMulti, Mettler Toledo, Columbus, OH, USA). The measurements were performed in triplicate.

#### 4.3.3. Flowability of the Gels

The flowability properties of the PSGs were determined to evaluate their pill-carrying ability through the pharynx and esophagus. We performed two in vitro tests, the tilted-aluminum-plate test, and the tablet-through-a-tube test to simulate gel movement during the swallowing process.

##### Gel Flow on a Tilted Aluminum Plate

**Figure 11:**
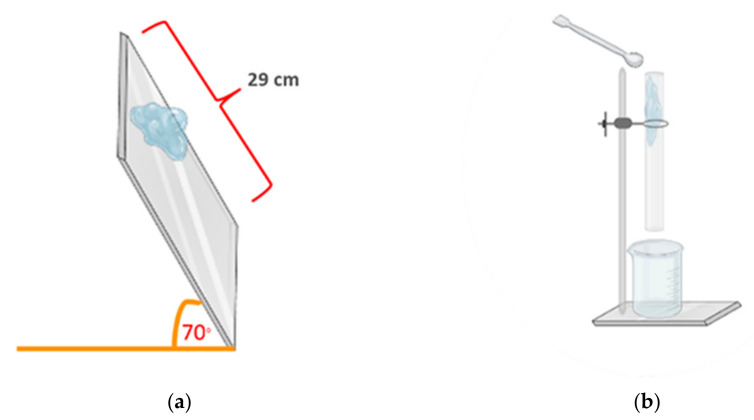
Schematic of (**a**) the tilted-aluminum-plate test and (**b**) the tablet-through-a-tube test.

The PSGs prepared from different polymer types and concentrations for 7.5 mL were used for the flow test. The test method was adapted from Marwa A. Malouh and co-workers [34]. We placed the gel samples on the top of a tilted (70°) aluminum plate (20 cm × 29 cm), as illustrated in Figure 11a. We recorded the distance and time of gel movement to calculate the flow speed. The tests were performed in triplicate.

##### Tablet Carrying through a Tube

The PSGs displaying a high flow speed in the tilted-aluminum-plate test were selected for the tablet-through-a-tube test. We designed this test, which was adapted from the commercial product testing method [35], to simulate gel movement in the human esophagus. We immersed a theophylline tablet (model drug) in 5 mL of the PSG and poured it through a clear rubber tube with a diameter of 2 cm and length of 30 cm in a vertical position, as depicted in Figure 11b. The distances and time of gel movement were recorded to calculate the flow speed. The tests were performed in triplicate.

#### 4.3.4. PSG Effect on Tablet Disintegration

**Figure 12:**
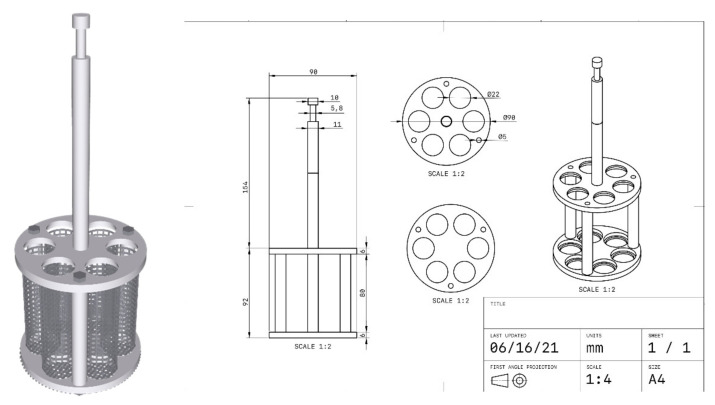
Modified tablet disintegration tester.

Since gels blocked the mesh of the basket of the disintegration tester apparatus, we could not follow the general chapter 701 of the U.S. Pharmacopeia (USP40) [31] to assess how PSGs affect tablet disintegration. Therefore, the applied disintegration apparatus was adapted from Alejandro Ruiz-Picazo [36] to evaluate the disintegration time, as shown in Figure 12. The theophylline tablet was immersed in 5 mL of PSG, which was then poured into the adapted basket. The basket dipping rate was 30 ± 1 stroke/min, and the medium was purified water at a controlled temperature of 37 ± 2 °C.

#### 4.3.5. PSG Effect on Drug Dissolution

The effect of the PSGs on drug dissolution was assessed following the theophylline tablet monograph from USP40 [31]. The dissolution profiles of plain theophylline tablets and the theophylline tablets covered with the different PSGs were determined using USP dissolution apparatus II (Erweka, Langen, Germany) equipped with paddles operated at 50 rpm. We poured 900 mL of purified water (pH 6.4), as the dissolution media, into the glass vessel; assembled the apparatus; and equilibrated the dissolution medium to 37 ± 0.5 °C. We took fluid test samples (5 mL) at 10, 30, 45, 60, and 120 min. Each time we took a sample, we added 5 mL of purified water to maintain the sink condition. We quantified theophylline release using a UV–VIS spectrophotometer (2J1-0004, Hitachi, Tokyo, Japan) at 272 nm and calculated the drug concentrations using a standard curve. We conducted each in vitro release study in triplicate and plotted the mean values of the percentage of drug release against time.

### 4.4. Statistical Analysis

We performed an analysis of variance and Student’s *t*-test using Microsoft Excel version 2102 for Microsoft 365 to determine any significant difference between each result. The dissolution profiles were analyzed between theophylline tablets covered and not covered by the PSG using the difference factor (*f*_1_) and the similarity factor (*f*_2_) to determine the effect of covering gel on the in vitro drug release. We computed *f*_1_ using the following equation [37]:

(1) $$f_{1}=\left\{\frac{\sum_{t=1}^{n}\left|R_{t}-T_{t}\right|}{\sum_{t=1}^{n}R_{t}}\right\}\times 100$$

where *n* is the number of time points, *R_t_* is the dissolution value of the reference (pre-change) batch at time *t*, and *T_t_* is the dissolution value of the test (post-change) batch at time *t*. We used the mean dissolution profiles from both profiles at each time interval to calculate the fit factors. We calculated the similarity factor (*f*_2_) as follows [38]:

(2) $$f_{2}=50\times \log\left\{{\left[1+\frac{1}{n}\overset{n}{\underset{j=1}{\sum }}{\left(R_{f}-T_{f}\right)}^{2}\right]}^{-0.5}\times 100\right\}$$

where *n* is the number of time points, and *R_j_* and *T_j_* are the percentages of reference and test product, respectively, released into the dissolution medium at time *j*.

## Figures and Tables

**Table 1:** Disintegration time of the theophylline tablets covered with the PSG prepared from carboxymethyl cellulose (CMC), low molecular weight chitosan (LMCS), and high molecular weight chitosan (HMCS), offering proper viscosity and flow ability.

Ingredient	Formulation (% *w*/*w*)		
	CMC Gel	LMCS Gel	HMCS Gel
Carboxymethyl cellulose	4	-	-
Low molecular weight chitosan	-	5	-
High molecular weight chitosan	-	-	6.25
Glycerin	1	6	4
Purified water	qs	qs	qs
Tablet disintegration time (s)	10.10 ± 1.37	10.72 ± 4.19	6.70 ± 3.22

**Table 2:** *f*_1_ (similarity factor) and *f*_2_ (dissimilarity factor) of the theophylline tablets covered with several gel type compared with the plain tablet.

	*f*_1_: Similarity Factor (Limit: 50–100)	*f*_2_: Dissimilarity Factor (Limit: 0–15)	Dissolution Difference Factor (*f*_1_ − *f*_2_)
Theophylline tablet with reference gel product	45	12	33
Theophylline tablet with carboxymethyl cellulose	37	19	18
Theophylline tablet with low molecular weight chitosan	43	13	30
Theophylline tablet with high molecular weight chitosan	40	16	24
